# High umbilical cord blood lead levels and “calabar chalk” consumption amongst pregnant women in two hospitals in Cameroon

**DOI:** 10.11604/pamj.2019.33.109.13999

**Published:** 2019-06-13

**Authors:** Brice Nguedia Vofo, Gaelle Vanelssa Fotsing Ngankam Vofo, Beatrice Ambo Fonge, Dickson Shey Nsagha, Thomas Obinchemti Egbe, Jules Clement Nguedia Assob

**Affiliations:** 1 Department of Obstetrics and Gynaecology, Faculty of Health Sciences, University of Buea, South West Region, Cameroon; 2 Department of Botany, Faculty of Science, University of Buea, South West Region, Cameroon; 3 Department of Public Health and Hygiene, Faculty of Health Sciences, University of Buea, South West Region, Cameroon; 4 Department of Medical Laboratory Sciences, Faculty of Health Sciences, University of Buea, South West Region, Cameroon

**Keywords:** “Calabar chalk”, pica, lead, umbilical cord, pregnancy

## Abstract

**Introduction:**

“Calabar chalk” is a lead-laden pica mostly consumed by pregnant women worldwide as a remedy for morning sickness. This clay material has been shown to have lead levels of up to 40mg per kilogram. Meanwhile blood lead levels, even at doses less than 10μg/dl will be toxic to humans and even worse-off to the fetus as it crosses the placenta. We, therefore, sort to determine the prevalence of “Calabar chalk” consumption amongst pregnant women and if it translates to higher umbilical cord blood lead levels.

**Methods:**

We carried out a cross-sectional study by prospectively and consecutively enrolling 300 pregnant women from December 2014 through February 2015. A questionnaire was administered to ascertain “Calabar chalk” consumption. The levels of lead in the umbilical cord blood of 51 participants of each group of those who consumed and didn't consume “Calabar chalk” were measured by spectrometry and compared using the T-test (p<0.05).

**Results:**

The prevalence of “Calabar chalk” consumption was 43.33%. This was mostly consumed during pregnancy only (46.34%), with higher rates observed amongst primigravidas. The mean umbilical blood lead levels amongst those who consumed and those who did not consume “Calabar chalk” was 39.19μg/dl and 25.33μg/dl respectively (P=0.111).

**Conclusion:**

The prevalence of “Calabar chalk” consumption was high in the pregnant women population. The overall umbilical cord blood lead levels were extremely high in both consumers and non-consumers. We recommend health education and chelation therapy to be considered.

## Introduction

“Calabar chalk”, often known as calabash clay, “Nizu”, “Poto”, “Calabar Stone”, “Ndom”, “Mabele”, “Argile” or “La Craie”, is a non-food substance commonly found in West Africa and is widely consumed for various reasons according to traditional believes [1]. It has been shown to contain lead, which is a well-established toxic substance to our organism. The variety commonly found in our community is the non-salted, shapeless pellets, with an average weight of 35grams obtainable at the cost of FCFA 25. Women who patronize the consumption of this non-food substance, report it as being an addiction, or consume it as a treatment for diarrhea; others say it relieves dyspepsia. Pregnant women, who should be the most concerned about their nutrition, also consume this “Calabar chalk” in large quantities. In Georgia, Grisgby and colleagues reported the consumption of “Calabar chalk” by pregnant women as a remedy for morning sickness [2]. In our community, many pregnant women also report consumption of “Calabar chalk” in the second and third trimesters to relieve dyspepsia. According to an analysis done by energy dispersive x-ray fluorescence spectroscopy (EDXRF) the mean concentration of lead in “Calabar chalk” was approximately 40 mg/kg [3]. In the human body, about 95% of absorbed lead is mainly transferred bound on the surface of red blood cells while about 5% is bound to plasma proteins. About 95% of this lead will be stored in the bones as lead-phosphate complexes [4]. Continuous exposure will easily cause tissue accumulation to toxic levels because lead is poorly excreted from the bodies. The main route of excretion is by way of the kidneys, where it is normally excreted at less than 0.5μmol/l. The physiology of pregnancy is also postulated to remobilize the stored lead from the skeletal tissues to the bloodstream [5]. Thus, the fetus is exposed to a double portion of lead; that which is consumed during pregnancy and that remobilized from the skeletal tissues.

It has been shown that there is a strong correlation between maternal and umbilical cord blood lead levels, indicating the easy transfer of lead from mother to fetus [4]. This transfer has also been shown to begin as early as the 12^th^ week of gestation when the central nervous system is still very vulnerable, the blood-brain barrier still very permeable and the fetus has less bone tissue for the sequestration of lead [4]. There seem to be no blood lead levels that could be considered safe. Since 1991, the Centre for Disease Control and Prevention (CDC) recommends blood lead levels of less than 10ug/dl in the pediatric population [6]. More recent studies showed that exposure of the fetus, even to these low levels of lead(<10μg/dl), could cause adverse fetal outcomes [5]. These fetal outcomes range from prematurity, iron deficiency anemia, low birth weight and miscarriages [5] to effects on the neurocognitive development of the fetus causing a substantial decrease in IQ (intelligence quotient) [4]. This eventually affects the child causing behavioral disorders such as violence [7, 8], elevated school drop-out rate, a potential link to criminal behavior [9] and juvenile delinquency [10]. To conclude, “Calabar chalk” is widely consumed, especially by pregnant women, meanwhile, this substance has been shown to contain alarming levels of lead that readily crosses the placenta as early as the 12^th^ week of pregnancy, to greatly impair the normal development of the vulnerable fetus. We, therefore, aimed to determine the prevalence of consumption of “Calabar chalk” in a population of pregnant women attending the Yaoundé Central and Buea Regional Hospitals; and to determine the mean levels of lead in the umbilical cord at delivery and its association with “Calabar chalk” consumption.

## Methods

This was a hospital-based observational cross-sectional analytic study, carried out in the Yaoundé Central and Buea Regional Hospitals which are referral hospitals in the regions of Yaoundé and Buea respectively and beyond. Yaoundé is the Political capital of Cameroon and its central hospital registers the highest number of births in the country. The study population was made up of pregnant women received in labor at the maternities of the Yaoundé central and Buea regional hospitals during the study period. The selection of study participants was done by convenience and consecutive sampling method. The sample size was determined using the formula, where “n", the estimated sample size; “z” as the critical z score based on the desired degree of confidence; “p” being the prevalence. P, the pre-study estimate was taken to be 25%. This is according to a study that described the prevalence of pica consumption among pregnant women in Sub-Saharan Africa to be 25.7% [11]. Finally, a value of 300 was obtained to represent the target population and was used to calculate the prevalence, amongst which 102 umbilical blood lead levels where analyzed. Participants who gave written consent were included, while women with sickle cell anemia, diabetes, and cardiovascular diseases were not included. Ethical and administrative approvals were first obtained from the Institutional Review Board of the Faculty of Health Sciences, University of Buea and the Hospital Directors, respectively.

Potential participants were approached during labor in these hospitals' labor rooms. Participants who could read and who showed interest to participate were made to read the consent form, while participants who could not read were given a comprehensive explanation of the content of consent form by the primary investigator. Participants were given ample time to ask questions before giving a verbal consent at this stage and within 18hrs after delivery, they were approached again for confirmation of their initial verbal consent by signing the consent form. After obtaining informed consent, the participants were interviewed by the principal investigator using a structured questionnaire designed for this purpose, only then could the participant's data be incorporated in the study. The principal investigator assisted in monitoring the progression of labor. After delivery, he collected 5mls of blood from the umbilical cord, specifically in the umbilical artery, as it is more representative of the fetal blood wearing protective sterile gloves [12]. The umbilical artery has a smaller lumen, thicker wall, and contains less blood than the umbilical vein. The blood was poured into a heparinized tube and stored at room temperature with no risk of variability [13]. The analysis was done using an Atomic Absorption spectrometer in the Laboratory of Inorganic Chemistry of the University of Dschang, where the principal investigator, had been previously trained. In the laboratory, 7.5ml of concentrated HN03 acid and 2.5ml of concentrated HCL acid was added to 1ml of blood and heated at a moderate temperature of 80-90^0^C until all the Nitrous vapors were emitted. Then the residue was made to reach 50ml using distilled de-ionized water. At this stage, our sample was ready for lead analysis using the atomic absorption spectrophotometer. After data collection, the data was cross-checked for errors before entry into a personal computer. Data were analyzed and a test of statistical significance was performed using Epi-Info version 3.5.3 software.

**For objective 1:** which was to determine the prevalence of consumption of “Calabar chalk” in a population of pregnant women attending the Yaoundé Central and Buea Regional Hospitals, the outcome variable was consumption of “Calabar chalk” either before and during pregnancy or during pregnancy only. Prevalence was computed as the number of women who consumed “Calabar chalk” during pregnancy divided by the total number of women recruited in the study.

**For objective 2:** we were to determine the mean levels of lead in the umbilical cord at delivery and its association with “Calabar chalk” consumption. The predictor variable was “Calabar chalk” consumption either before or during pregnancy, while the outcome variable was the levels of lead in the umbilical cord blood. The mean (and Standard Deviation) level of lead was estimated amongst 51 women who consumed “Calabar chalk” and in 51 women who didn't consumed. The two means were then compared using the t-test, with a p-value <0.05 being considered statistically significant.

## Results

In this study we approached 387 pregnant women, we excluded 43 women who did not meet the inclusion criteria and 44 women did not consent. We finally included 300 women in the study and among these women, we tested 102 umbilical blood lead levels.

**General characteristic of the study participants:** the age of the study participants ranged from 15 to 42 years with a mean age of 27.25 ± 5.9 years. The single, divorced and widowed women were all grouped as unmarried and 196 (65.33%) women were unmarried. The occupations of the participants were classified as either: unemployed, skilled jobs or unskilled jobs. The skilled jobs were considered as those that require more of mental activity than physical activity and the unskilled jobs were considered as those that require more physical activity than mental activity. Of the 300 participants, more than half, 160 (53.33%), were unemployed. The General Characteristics of the participants are summarized in Table 1.

**Table 1:** General characteristics of the 300 study participants

Characteristic		Total N=300	Percentage
**Age group**	15-20 Years	36	12.00%
	21-25 Years	92	30.67%
	26-30 Years	78	26.00%
	31-35 Years	64	21.33%
	36-40 Years	26	8.67%
	41-45 Years	4	1.33%
**Marital status**	Married	104	34.67%
	Unmarried	196	65.33%
**Occupation**	Unemployed	160	53.33%
	Unskilled labour	80	26.67%
	Skilled labour	60	20.00%

**Figure 1:**
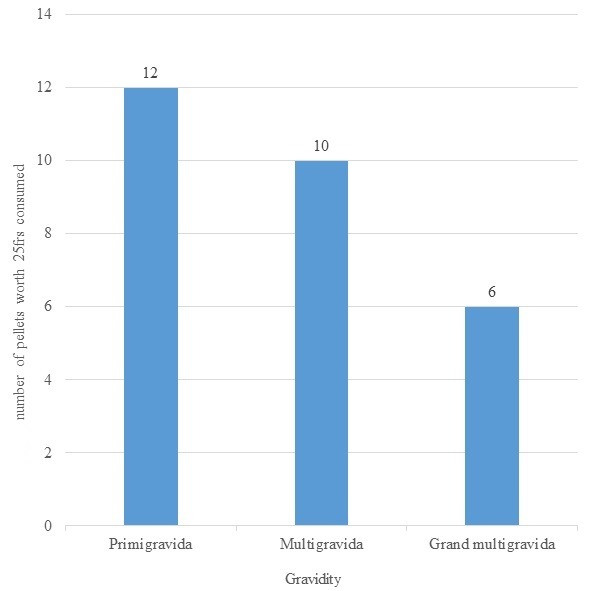
Mean number of pellets of “calabar chalk” worth 25 FCFA consumed each week as per their gravidity

**Amount of “Calabar chalk” consumption as per the gravidity of participants:** primigravidas reported the highest frequency and quantity of “Calabar chalk” consumption as shown in Figure 1.

**Prevalence of “Calabar chalk” Consumption:** of the 300 participants recruited in the Yaoundé Central and Buea Regional Hospitals, the prevalence of “Calabar chalk” consumption (both those who consumed before and during pregnancy and those who consumed during pregnancy only) was 43.33%,(95% CI 37.6-49.1%).

**Mean umbilical blood lead levels amongst those who consumed “Calabar chalk” and those who did not:** we analyzed 102 umbilical cord blood samples, 51 from each group of those who consumed and didn't consume “Calabar chalk” were measured by spectrometry. The lead levels ranged from 9μg/dl to 92μg/dl, with a mean value of 28.62μg/dl ± 18.431μg/dl. The mean lead levels amongst those who consumed “Calabar chalk” was 31.19ug/dl ± 18.55ug/dl and amongst those who did not was 25.33ug/dl ± 17.95ug/dl (P= 0.111).

## Discussion

In this study, we looked at the prevalence of “Calabar chalk” consumption, and its association with blood lead levels amongst pregnant women in the Yaoundé central and Buea Regional hospitals. Amongst the 300 pregnant women recruited, we found that 43.33% (130/300) of the pregnant women consumed “Calabar chalk”. Mean umbilical cord blood lead levels were 31.19 μg/dl and 25.33 μg/dl for those who consumed and for those who did not consume “Calabar chalk” respectively (p= 0.111). The prevalence of “Calabar chalk” consumption during pregnancy in our study at the Central and Buea regional hospitals (43.33%) was lower than the 50% reported by Sule and Madugu amongst pregnant women consuming Pica in Zaria, Nigeria [14]. The higher prevalence in this study could be because they considered Pica in general, and not “Calabar chalk” precisely. However, our findings are in accordance with that reported by Edward *et al* [15] who described the prevalence of pica consumption by pregnant women to range from 8% to 64%, depending on the population studied. The mean umbilical blood lead levels amongst those who consumed “Calabar chalk” (31.19μg/dl) was higher than that amongst those who did not (25.33μg/dl), although the difference was not statistically significant (p=0.11). This is surprising, given that “Calabar chalk” has been shown to contain lead [3]. But the generally high levels of lead in both groups is quite alarming, as we observed umbilical blood lead levels ranging from 9μg/dl to 92μg/dl, and considering the fact that during breastfeeding the neonatal blood lead levels could further increase as mothers have been shown to transfer about 3% of their blood lead to their babies during breastfeeding [16].

Our study appears to be the first to have looked at blood lead levels in Cameroon, but this overall high blood lead levels observed are similar to that which was observed amongst children less than 5 years in a study conducted in Nigeria. In this study, the blood lead levels ranged from 8μg/dl to 332μg/dl [17]. This could imply that there are so many other sources of lead intoxication yet to be identified. Some studies carried out in Cameroon have identified some sources of lead exposure, amongst which the most alarming was that recently reported by Weidenhamer and colleagues in 2014, that described high levels of lead exposure from our most widely used aluminum cookware, especially when heated [18]. Fonge and colleagues also described high lead levels in the bones and flesh of fishes in the Douala Estuary [19]. Plants have also been shown to bio-accumulate lead if grown on soils containing it [20]. A lot of commercial farming is being done along the fertile soils of Mount Cameroon, which might be rich in lead given that being a heavy metal found in the earth's crust beneath, could have been propelled out during volcanic eruptions, and can now being bio-accumulated by plants consumed by humans. Furthermore, our plumbing systems, if made of lead-containing metals, could also greatly expose us to lead intoxication. Blood lead levels even less than 10μg/dl have been associated with 6 points loss in Intelligence quotient [21], amongst other serious toxic effects and CDC recommends that mothers with blood lead levels greater than 40μg/dl, should not even breastfeed their babies [16]. Thus, these findings call for immediate public health attention. A possible solution could be the use of chelation therapies during pregnancy. The CDC recommends chelation therapy during pregnancy if Blood lead levels are over 45μg/dl and agents such as calcium disodium EDTA and Succimer have been tried during the 2^nd^ and 3^rd^ trimesters [16], as data on the reproductive risk associated with the use of these chelation agents during the first trimester are sparse.

**Study limitations** dependence on the memory of post-partum women which could not be very accurate, so our findings might not be the true picture of the actual situation as far as quantity and frequency of “Calabar chalk” consumption was concerned. The atomic absorption spectrophotometer used in the quantification of the lead in blood has a relatively high detection limit of approximately 10μg/dl but this might not have affected our study, as we mostly had lead levels above that threshold. There is a potential for selection bias here since our recruitment was restricted to patients attending public hospitals with a relatively lower cost of treatments. This group tends to be of lower socioeconomic status and may have different frequencies of “Calabar chalk” consumption. Consequently, this might have caused an over-estimation of the actual prevalence of this practice in Cameroon.

## Conclusion

The following conclusions can be drawn from this study: the prevalence of “Calabar chalk” consumption during pregnancy amongst pregnant women attending the Yaoundé Central and Buea Regional hospitals was 43.33%. Amongst pregnant women, primigravidas are the highest consumers of “Calabar chalk”. The mean umbilical cord blood lead level was higher amongst those who consumed “Calabar chalk”, though not statistically significantly different. Both groups had extremely high umbilical blood lead levels. We recommend health education and the use of chelation therapies to be considered amongst pregnant women.

### What is known about this topic

- That the prevalence of Pica consumption ranges from 8% to 64% depending on the population studied;
- “Calabar chalk” contains high levels of Lead;
- It's known that lead crosses the placenta to the fetus during pregnancy.

### What this study adds

- That amongst pregnant women, primigravidas are the highest consumers of “calabar chalk";
- This study reveals overall high umbilical blood lead levels amongst pregnant women at birth;
- That “calabar chalk” does not significantly increase the umbilical blood lead levels of pregnant women who consumed it as compared to those who don't consume it.

## Competing interests

The authors declare no competing interests.
